# Clinical effectiveness of pneumococcal vaccination against acute myocardial infarction and stroke in people over 60 years: the CAPAMIS study, one-year follow-up

**DOI:** 10.1186/1471-2458-12-222

**Published:** 2012-03-22

**Authors:** Angel Vila-Corcoles, Olga Ochoa-Gondar, Teresa Rodriguez-Blanco, Antonia Gutierrez-Perez, Angel Vila-Rovira, Frederic Gomez, Xavier Raga, Cinta de Diego, Eva Satue, Elisabet Salsench

**Affiliations:** 1 Primary Care Service of Tarragona, EPIVAC Study Group, Institut Catalá de la Salut, Tarragona, Spain; 2 Primary Care Research Institute (IDIAP Jordi Gol) and research associate, Autonomous University of Barcelona (UAB), Barcelona, Spain; 3 Department of Laboratory and Microbiology, Hospital Joan XXIII, Tarragona, Spain; 4 Department of Laboratory and Microbiology, Hospital Santa Tecla, Tarragona, Spain

## Abstract

**Background:**

Conflicting results have been recently reported evaluating the relationship between pneumococcal vaccination and the risk of thrombotic vascular events. This study assessed the clinical effectiveness of the 23-valent polysaccharide pneumococcal vaccine (PPV23) against acute myocardial infarction and ischaemic stroke in older adults.

**Methods:**

Population-based prospective cohort study conducted from December 1, 2008 until November 30, 2009, including all individuals ≥ 60 years-old assigned to nine Primary Care Centres in Tarragona, Spain (N = 27,204 individuals). Primary outcomes were hospitalisation for acute myocardial infarction and/or ischaemic stroke. All cases were validated by checking clinical records. The association between pneumococcal vaccination and the risk of each outcome was evaluated by Multivariable Cox proportional-hazard models (adjusted by age, sex, influenza vaccine status, presence of comorbidities and cardiovascular risk factors).

**Results:**

Cohort members were followed for a total of 26,444 person-years, of which 34% were for vaccinated subjects. Overall incidence rates (per 1000 person-years) were 4.9 for myocardial infarction and 4.6 for ischaemic stroke. In the multivariable analysis, vaccination was associated with a marginally significant 35% lower risk of stroke (hazard ratio [HR]: 0.65; 95% confidence interval [CI]: 0.42-0.99; *p *= 0.046). We found no evidence for an association between pneumococcal vaccination and reduced risk of myocardial infarction (HR: 0.83; 95% CI: 0.56-1.22; *p *= 0.347).

**Conclusions:**

Our data supports a benefit of PPV23 against ischaemic stroke among the general population over 60 years, suggesting a possible protective role of pneumococcal vaccination against some acute thrombotic events.

## Background

The 23-valent polysaccharide pneumococcal vaccine (PPV23) is recommended for high-risk and older adults [1-3], although its effectiveness remains unclear [4,5]. Numerous studies have demonstrated that PPV23 provides considerable protection against invasive pneumococcal disease [2,3], while a possible protective effect against other clinically relevant outcomes as community-acquired pneumonia is controversial [4-6].

In recent years, considering that some studies have reported an increasing risk of thrombotic events among patients with pneumonia [7-9], it has been suggested that pneumococcal vaccination would protect patients from coronary and/or cerebrovascular attacks. However, three studies using health databases to evaluate the effectiveness of pneumococcal vaccination in preventing myocardial infarction and/or stroke have recently been published with conflicting results [10-12].

Thus, given a potential effect of pneumococcal vaccination on cardiovascular prevention could have immense clinical implications, prospective studies, which will determine whether the protective effect of vaccination is real or not are greatly needed.

In Catalonia, a region in the northeast of Spain with a population of seven million people, a publicly funded anti-pneumococcal vaccination programme for all individuals 60 years or older (with or without risk factors) began in October 2002. Since then, the PPV23 is offered when the patients came to the primary care centres during the annual influenza vaccination campaigns or in any other visit throughout the year [13]. Revaccination at 5 years is only recommended for people under 65 years.

Following this, we designed a large prospective cohort study, known as *CAPAMIS*, with the major aim of evaluating the potential role of the PPV23 in cardiovascular prevention among the general population over 60 years [14]. The *CAPAMIS *study was planned for 3-year follow-up. In this study, we have evaluated the clinical effectiveness of PPV23 in reducing the risk of hospitalisation for CAP, acute myocardial infarction and ischaemic stroke in a first-time analysis at one-year follow-up.

## Methods

### Design, setting and study population

Study design has been extensively described elsewhere [14]. In brief, this is a closed population-based prospective cohort study including 27,204 individuals 60 years or older assigned to nine primary care centers in the Health Region of Tarragona (a mixed residential-industrial urban area in the Mediterranean coast of Catalonia, Spain). The study was approved by the ethical committee of the Catalonian Health Institute (P09/49) and was conducted in accordance with the general principles for observational studies. In this first-time report, we analyse the primary end-points resulting from the first year of survey. Cohort members were followed since the start of the study (December 1, 2008) until the occurrence of any event, change in pneumococcal vaccination status, disenrollment from the primary care center, death, or until the end of first 12-month follow-up (November 30, 2009).

### Data sources

All participating primary care centers have a computerized clinical record system that includes administrative data, medical conditions, prescriptions, laboratory results and diagnosis associated with hospital and outpatient visits. This electronic clinical record system was used to classify cohort members by their pneumococcal vaccination status as well as to identify comorbidities or underlying conditions and establish baseline characteristics of the cohort at study entry. The hospital diagnosis discharge databases of the two reference hospitals in the study area (Joan XXIII and Santa Tecla), coded according to the International Classification of Diseases, 9th Revision, Clinical Modification (ICD-9) were used to identify study events.

### Outcomes

The primary outcomes were hospitalisation for community acquired pneumonia, acute myocardial infarction, ischaemic stroke and death from any cause. Outcomes were initially identified on the basis of listed ICD-9 diagnosis codes for pneumonia (480 to 487.0), myocardial infarction (410) and ischaemic stroke (433, 434, 436 and 437). Cases were only definitively included if, on conclusion of the medical record review, the physician reviewer (two specialist family physicians previously trained) verified the diagnosis according to criteria mentioned below.

Pneumonia was defined when a new radiological infiltrate was identified with one major criteria (cough, expectoration or fever) or two minor criteria (dyspnea, pleuritic pain, altered mental status, pulmonary consolidation on auscultation and leukocytosis) [14].

Acute Myocardial Infarction was defined as a detection of rise and/or fall in cardiac biomarkers together with at least one of the following: symptoms of ischaemia, ECG changes indicative of new ischaemia (new ST-T changes, new left bundle branch block and/or development of pathological Q waves) and/or imaging evidence of new loss of viable myocardium or new regional wall motion abnormality [15].

Ischaemic stroke was considered when a patient rapidly developed signs of focal or global disturbance of cerebral function lasting more than 24 h (unless interrupted by surgery or death), with no apparent nonvascular cause and a neuro image showing an ischaemic brain lesion [16].

### Vaccination history

Pneumococcal vaccination status was determined by a review of the primary care centers' electronic clinical records, which contain specially designated fields for pneumococcal and influenza vaccinations. We assumed that information in the computerized clinical records system (working since 1999) was complete, so a subject was considered as unvaccinated when a vaccination was not recorded. Cohort members were classified as vaccinated against pneumococcus if they had received at least one dose of PPV23 in the last 60 months before study start.

### Statistical analysis

Incidence rates were calculated as person-years, considering in the denominator the sum of the persons-time contributed to each individual during the study period. Baseline characteristics according to pneumococcal vaccination status were compared using Chi-squared test.

Cox proportional hazards models were used to assess the association between having received the pneumococcal vaccine and the time to the first outcome [17]. The final models were adjusted for significant and confounder variables.

The following variables were considered in all the initial models: age, sex, number of outpatient visits to family physician in 12-months before study start (< 3,3-5,6-9,≥10), influenza vaccination in prior autumn, history of coronary artery disease (myocardial infarction or angina), history of stroke, history of chronic heart disease (congestive heart failure, hypertensive heart disease, cardiomyopathy, valvulopathy, cardiac dilatation or ventricular hypertrophy), chronic pulmonary disease (chronic bronchitis, emphysema and asthma), hypertension, hypercholesterolemia, obesity, diabetes mellitus, smoking status (non-smoker, quit, current), alcoholism, chronic severe liver disease(chronic viral hepatitis, alcoholic hepatitis and cirrhosis), chronic severe nephropathy (nephrotic syndrome, renal failure, dialysis or transplantation), cancer (solid organ or haematological neoplasia), dementia and nursing-home residence. Age, sex and influenza vaccine status were judged epidemiologically relevant variables, being included in all the final models. The variables were time-invariant and defined at study entry.

The authors checked for confounders and multicolinearity among the independent variables. The proportional hazard assumptions were assessed by adding the covariate by time interactions to the model and plotting the scaled and smoothed Schoenfeld residuals obtained from main effects model, where possible. All results were expressed with 95% confidence intervals (CIs). Statistical significance was set at *p *< 0.05 (two-tailed). The analyses were performed using Stata/SE version 11.1 for Windows (StataCorp. LP).

## Results

The 27,204 cohort members were observed for a total of 26,444 person-years, of which 8,847 (33.5%) person-years had received PPV23 in prior 5 years.

Mean age of study subjects when study started was 71.7 (SD: 8.6) years-old and 44.6% were male. Vaccinated group were significantly older, had more outpatient visits, had a much higher proportion of influenza vaccination and had more comorbidities than the unvaccinated group (Table 1).

**Table 1:** Baseline Characteristics of 27,204 cohort members according to their Pneumococcal vaccination status before the study started

Characteristic	Unvaccinated^a^ (n = 18,223)	Vaccinated (n = 8,981)	*p *value^b^	Total N = 27,204
			
	No. (%)			
Age group, yrs^c^				
60-69	8522 (46.8)	3879 (43.2)	< 0.001	12401 (45.6)
70-79	5833 (32.0)	3451 (38.4)		9284 (34.1)
≥ 80	3868 (21.2)	1651 (18.4)		5519 (20.3)

Sex, Male	8074 (44.3)	4063 (45.2)	0.145	12137 (44.6)

No. of outpatient visits during previous 12 months^d^				
[0-2]	4886 (26.8)	1168 (13)	< 0.001	6054(22.3)
[3-5]	4183 (23.0)	2207 (24.6)		6390(23.5)
[6-9]	4338 (23.8)	2569 (28.6)		6907(25.4)
≥10	4816 (26.4)	3037 (33.8)		7853(28.9)

Influenza vaccination in previous Autumn	6997 (38.4)	7371 (82.1)	< 0.001	14368 (52.8)

History of coronary artery disease	1122 (6.2)	611 (6.8)	0.040	1733 (6.4)

History of stroke	808 (4.4)	486 (5.4)	< 0.001	1294 (4.8)

Chronic pulmonary disease	1421 (7.8)	742 (8.3)	0.183	2163 (8.0)

Chronic heart disease**^e^**	2084 (11.4)	1324 (14.7)	< 0.001	3408 (12.5)

Chronic liver disease	383 (2.1)	239 (2.7)	0.004	622 (2.3)

Chronic nephropathy	44 (2.4)	214 (2.4)	0.786	658 (2.4)

Diabetes mellitus	3713 (20.4)	2192 (24.4)	< 0.001	5905 (21.7)

Hypertension	9245 (50.7)	5304 (59.1)	< 0.001	14549 (53.5)

Hypercholesterolemia	6351 (34.9)	3611 (40.2)	< 0.001	9962 (36.6)

Obesity (BMI > 30)	5089 (27.9)	3150 (35.1)	< 0.001	8239 (30.3)

Smoking status				

Non-smoker	12855 (70.5)	5826 (64.9)	< 0.001	18681(68.7)

Quit	3479 (19.1)	2248 (25.0)		5727(21.1)

Current	1889 (10,4)	907 (10.1)		2796(10.3)

Alcoholism	655 (3.6)	340 (3.8)	0.429	995 (3.7)

Cancer	1297 (7.1)	703 (7.8)	0.035	2000 (7.4)

Immunosuppressive medication	772 (4.2)	425 (4.7)	0.061	1197 (4.4)

Immunocompromise^f^	1921 (10.5)	1005 (11.2)	0.104	2926 (10.8)

Dementia	535 (2.9)	266 (3.0)	0.905	801 (2.9)

Nursing-home residence	265 (1.5)	110 (1.2)	0.127	375 (1.4)

^a ^An amount of 6,179 individuals were classified as non-immunized because they had received the PPV23 more than 60 months before study start.

^b ^*p *values were calculated with chi-square test.

^c ^The mean ages of the unvaccinated and vaccinated subjects were 71.5 years (Standard Deviation, SD: 8.9) and 72.0 years (SD: 7.8) respectively. Mean age of overall study subjects was 71.7 years (SD: 8.6).

^d ^The mean number of outpatient visits during previous 12 months were 7.1 (SD: 6.7) in unvaccinated and 8.4 (SD: 6.3) in vaccinated subjects. Mean number of visits among the overall study population was 7.5 (SD: 6.6).

^e ^It includes congestive heart failure, hypertensive heart disease, cardiomyopathy, valvulopathy, cardiac dilatation or ventricular hypertrophy.

**^f ^**Immunocompromise was a composite variable defined by the presence of any one of the following: cancer (solid organ or haematological neoplasia), chronic severe nephropathy (nephrotic syndrome, renal failure, dialysis or transplantation), anatomical or functional asplenia, immunodeficiency (including AIDS), and long-term corticosteroid therapy (20 mg/day of prednisone) or another immunosuppressive medication.

During the 12-month follow-up, a validated episode of community acquired pneumonia was observed in 207 cases, a validated episode of acute myocardial infarction was observed in 130 cases and a validated episode of ischaemic stroke was observed in 121 cases. There was an incidence (per 1000 person-years) of 7.9 (95% CI: 6.9-9.0) for pneumonia, 4.9 (95% CI: 4.2-5.9) for myocardial infarction and 4.6 (95% CI: 3.8-5.5) for ischaemic stroke. Of the 27,204 cohort members, 840 died during study period (39 deaths from myocardial infarction/stroke). All-cause mortality rate was 31.8 per 1000 person-years (95% CI: 29.7-34.0), whereas specific mortality rates was 1.5 per 1000 (95% CI: 1.1-2.0) for myocardial infarction/stroke.

In the unadjusted analysis, we observed 63 episodes of pneumonia in 8,824 vaccinated person-years (7.1 per 1000 person-years) compared with 144 events in 17,546 unvaccinated person-years (8.2 per 1000 person-years). For myocardial infarction, there were 41 episodes of myocardial infarction in 8,830 vaccinated person-years (4.6 per 1000 person-years) compared with 89 events in 17,565 unvaccinated person-years (5.1 per 1000 person-years). For stroke, there were 30 events in 8,835 vaccinated person-years (3.4 per 1000 person-years) compared with 91 events in 17,559 unvaccinated person-years (5.2 per 1000 person-years). The unadjusted all-cause mortality rates were 26.1 and 34.6 per 1000 person-years among vaccinated and unvaccinated subjects, respectively.

In the multivariable analyses, despite vaccinated people had lower incidence rates than unvaccinated people, we found no evidence for an association between pneumococcal vaccination and risk of pneumonia (adjusted hazard ratio [HR]: 0.85; 95% CI: 0.62-1.15; *p *= 0.287) or myocardial infarction (adjusted HR: 0.83; 95% CI: 0.56-1.22; *p *= 0.347), but vaccination emerged significantly associated with a reduced risk of ischaemic stroke (adjusted HR: 0.65; 95% CI: 0.42-0.99; *p *= 0.048).

Although vaccinated subjects had lower all-cause mortality rates than unvaccinated subjects in the unadjusted analysis, vaccination was not associated with reduced risk of all-cause mortality in the multivariable analysis (adjusted HR: 0.88; 95% CI: 0.75-1.03; *p *= 0.118).

Table 2 shows the values of unadjusted and adjusted analyses for the different outcomes. The covariate pneumococcal vaccine has proportional hazards in all the models. Footnotes in table indicate those predictor variables statistically significant (*p *< 0.05) or confounders in the final multivariable models.

**Table 2:** Incidence and Risk of hospitalization for community acquired pneumonia (CAP), acute myocardial infarction (AMI), ischaemic stroke and death from any cause among patients 60 years or older in relation to pneumococcal-vaccination status^a^

	CAP	AMI	Ischaemic Stroke	Death from any cause
Number of event				
Vaccinated	63	41	30	231
Unvaccinated	144	89	91	609

Unadjusted incidence rate per 1000 person-years				
Vaccinated	7.1 (5.6-9.1)	4.6 (3.4-6.3)	3.4 (2.4-4.9)	26.1 (23.0-29.7)
Unvaccinated	8.2 (7.0-9.7)	5.1 (4.1-6.2)	5.2 (4.2-6.4)	34.6 (32.0-37.5)

Unadjusted hazard ratio for all subjects	0.87	0.91	0.66	0.75
(95% CI)	(0.65-1.17)	(0.63-1.32)	(0.43-0.99)	(0.65-0.88)
p value	0.368	0.637	0.046	< 0.001

Age, gender adjusted hazard ratio	0.87	0.90	0.66	0.79
(95% CI)	(0.65-1.17)	(0.62-1.30)	(0.44-1.00)	(0.68-0.91)
p value	0.354	0.582	0.049	0.002

	^b^	^c^	**^d^**	**^e^**
Multivariate hazard ratio	0.85	0.83	0.65	0.88
(95% CI)	(0.62-1.15)	(0.56-1.22)	(0.42-0.99)	(0.75-1.03)
p value	0.287	0.347	0.048	0.118

^a ^Hazard ratios were for vaccinated subjects as compared with unvaccinated subjects.

^b ^Adjusted for age, sex, number of outpatient visits in prior year, influenza vaccination in prior year, chronic pulmonary disease, chronic heart disease, smoking and nursing-home resident.

^c ^Adjusted for age, sex, number of outpatient visits in prior year, influenza vaccination in prior year, history of coronary artery disease, chronic heart disease, diabetes mellitus, hypercholesterolemia, smoking (confounder) and nursing-home resident.

^d ^Adjusted for age, sex, number of outpatient visits in prior year, influenza vaccination in prior year, history of coronary artery disease, history of stroke, smoking (confounder) and nursing-home resident.

^e ^Adjusted for age, sex, number of outpatient visits in prior year, influenza vaccination in prior year, chronic pulmonary disease, chronic heart disease, diabetes mellitus, cancer, chronic nephropaty, dementia, hypertension, hypercholesterolemia, obesity, smoking, and nursing home-resident.

In a separate analysis including only immunocompetent people n = 24,278 persons, the results did not substantially vary. Among these subjects, with multivariable adjustment, pneumococcal vaccination did not emerge significantly effective against pneumonia (adjusted HR: 0.84; 95% CI: 0.59-1.19; *p *= 0.319), myocardial infarction (adjusted HR: 0.83; 95% CI: 0.55-1.25; *p *= 0.363) or death from any cause (adjusted HR: 0.84; 95% CI: 0.68-1.02; *p *= 0.083), but it was associated with a significant reduction in the risk of ischaemic stroke adjusted HR: 0.63; 95% CI: 0.40-0.99; *p *= 0.049).

Analyses restricted to persons with possible immunocompromise (n = 2,926 subjects) showed, in general, poor results. Among these subjects, we did not observe any significant protective effect of vaccination against pneumonia (adjusted HR: 0.92; 95% CI: 0.47-1.81; *p *= 0.811), myocardial infarction (adjusted HR: 0.81; 95% CI: 0.25-2.66; *p *= 0.732), ischaemic stroke (adjusted HR: 0.75; 95% CI: 0.23-2.41; *p *= 0.628) or death from any cause (adjusted HR: 0.99; 95% CI: 0.75-1.30; *p *= 0.941).

Analyses excluding people with history of prior coronary artery disease (n = 1,733 persons) or stroke (n = 1,294 persons), did not substantially vary the results.

In the analyses including exclusively 25,471 subjects without history of coronary artery disease, we observed 30 episodes of myocardial infarction in 8,236 vaccinated person-years (3.6 per 1000 person-years) compared with 48 events in 16,510 unvaccinated person-years (2.9 per 1000 person-years). This means that pneumococcal vaccination was not associated with significant reductions in the risk of myocardial infarction in the unadjusted analysis (HR: 1.25; 95% CI: 0.79-1.98; *p *= 0.344) neither in the multivariable analysis (adjusted HR: 1.12; 95% CI: 0.69-1.82; *p *= 0.660).

Among the 25,910 subjects without history of prior stroke, we observed 13 episodes of ischaemic stroke in 8,366 vaccinated person-years (1.6 per 1000 person-year) compared with 60 events among 16,810 unvaccinated person-years (3.6 per 1000 person-years), which pointed to a significant reduction in the risk of ischaemic stroke among vaccinated subjects in the unadjusted analysis (HR: 0.43; 95% CI: 0.24-0.79; *p *= 0.006). Multivariable analysis confirmed this association (adjusted HR: 0.46; 95% CI: 0.25-0.85; *p *= 0.013).

In the total study population, according to incidence data among vaccinated and unvaccinated subjects for the different outcomes, number needed to vaccinate for preventing one case of ischaemic stroke was 560 vaccinations (95% CI: 295 to 5,649). If we consider pneumonia and myocardial infarction, although the results did not reach statistical significance, number needed to vaccinate were 938 and 2,365 vaccinations, respectively (Table 3).

**Table 3:** Numbers needed to vaccinate for preventing one case of community acquired pneumonia, acute myocardial infarction or ischaemic stroke by pneumococcal vaccination in people 60 years or older

Outcome	Attributable Risk per 1000 person/year		Number Need to Vaccinate	
	
	AR^a^	95% CI^b^	NNV^c^	95% CI (lower to higher^d^)
**Community acquired pneumonia**	1.067	-1.139 to 3.327	938	306 to -878

**Acute myocardial infarction**	0.423	-1.341 to 2.187	2,365	458 to -746

**Ischaemic Stroke**	1.788	0.177 to 3.399	560	295 to 5649

^a ^Attributable Risk = incidence rate difference between unvaccinated and vaccinated subjects.

^b ^CI denotes confidence interval.

^c ^NNV is the number needed to vaccinate for preventing one case and is estimated as 1/AR.

^d ^The negative numbers in the high limit of confidence interval indicate that NNV tends to infinite and it is not statistically significant.

## Discussion

We undertook a large prospective population-based study to evaluate the controversial effectiveness of the PPV23 against myocardial infarction and stroke. To our knowledge, this is the first prospective study using validated clinical data that provides population based assessment of pneumococcal vaccination effectiveness against cardiovascular events. Although we did not conduct a randomized controlled trial, the large size of our study population together with adjustment for important covariables in the multivariable analysis, provides an adequate basis for assessing this major public health issue in the general population over 60 years (main target population where PPV23 is recommended).

As main finding, our data shows a marginally significant reduction in the risk of ischaemic stroke, suggesting a possible protective role of vaccination against some acute thrombotic events. Considering the total study population, pneumococcal vaccination was associated with a 35% (95% CI: 1% to 58%) reduction in the adjusted risk of ischaemic stroke. This result seems to be robust considering that this significant protective effect remains when people with history of prior stroke are excluded from the analysis. The observed protective effect of vaccination against myocardial infarction (17%) was weaker and did not reach statistical significance (-22% to 44%).

Prior studies that have tried to examine this question have reported conflicting results [10-12,18]. An earlier case-control study that included 335 case patients with myocardial infarction and 199 control subjects reported a non significant protective effect of PPV23 against myocardial infarction (odds ratio [OR]: 0.89 (95% CI: 0.60-1.33) [18]. In a Canadian hospital-based case-control study, 999 case patients who had been admitted for treatment of myocardial infarction were compared with 3,996 control patients reporting that cases were less likely than controls to have received PPV23 (7.1% *vs *11.6%; adjusted OR: 0.53; 95% CI: 0.40-0.70) [10]. Importantly, the study population only included persons at risk for myocardial infarction (persons with hypertension, diabetes mellitus and/or dyslipidemia) and "healthy user" bias possibly occurred in selecting controls [19,20].

In a recent retrospective large cohort study involving 84,170 men aged 45 to 69 years in California, Tseng et al. [11] examined the relationship between pneumococcal vaccination and risk of myocardial infarction and stroke, concluding that receipt of pneumococcal vaccine was not associated with subsequent reduced risk of both events. However, some major methodological distinctions may explain the different results observed in the present study and the Tseng's report. This study used validated clinical data to establish outcome events rather than billing data used in Tseng's report. In addition, this study includes all population over 60 years, whereas the study by Tseng et al. only included men 45-69 years-old without prior history of coronary artery disease or stroke. In addition, as it was noted,[21,22] it is not clear whether the protective effect of pneumococcal vaccination against vascular events in individuals aged 65 years or older (main target group for vaccination) was properly assessed in Tseng's report given that 78% of sample size were men less than 65 years-old. In fact, the adjusted Hazard Ratios for acute myocardial infarction (HR: 0.89; 95% CI: 0.80-1.01; *p *= 0.10) and stroke (HR: 0.85; 95% CI: 0.70-1.03; *p *= 0.10) observed in Tseng's report, although not reaching conventional significance levels, suggested that there might be a protective effect of pneumococcal vaccination against vascular events in people over 65 years-old [11,22].

In other large observational study using health databases in Hong Kong, Hung et al. [12] have recently reported significant lower risks of acute myocardial infarction (HR: 0.52; 95% CI: 0.38-0.71) and ischaemic stroke (HR: 0.67; 95% CI: 0.54-0.83) among elderly persons who received dual vaccination with PPV23 and influenza vaccine. Our estimates of protective effect of vaccination against ischaemic stroke is essentially similar to data observed in the Hung's report.

Multiple mechanisms could contribute to the potential cardiovascular protective effect of pneumococcal vaccination. Possible direct effects include a reduction risk for pathogenic cross reaction antibodies induced by persistent pneumococcal antigens. Animal experiments have shown that pneumococcal vaccination reduces the extent of atherosclerotic lesions, and it has been hypothesized that antibodies directed against pneumococus would also cross react with oxidized low-density lipoprotein and impede the formation of foam cells [23]. The role of acute infection in triggering acute coronary or cerebrovascular events would be complex and multifactorial (e.g., increased inflammatory activity, prothrombotic conditions, and biomechanical stress on the arteries, disrupting and triggering thrombosis in a preexisting advanced artery lesion) [24].

With regard to community acquired pneumonia, controversy about PPV efficacy against pneumonia exists [4-6]. The last Cochrane Review Reported a pooled vaccine efficacy of 29% (95% CI: 3% to 48%) against all-cause pneumonia. Our results showing a non-statistically significant 15% reduction in the adjusted risk of pneumonia among vaccinated subjects fits with data reported in Cochrane review and other meta-analysis and they do not exclude the possibility of a little, but non insignificant effect against pneumonia.

A major strength of this study is that it was population-based and included all target people for pneumococcal vaccination in a well defined geographical area. Other important strengths were the prospective design, the validation of outcome events by checking clinical records that protects against biases related to recall and the use of survival analysis methods to estimate vaccine effectiveness adjusted by important covariables such as age, sex, influenza vaccine status, presence of main comorbidities and cardiovascular risk factors. Two possible confounding factors (dietary habits and physical activity) were not measured in the present study. However, given multicolinearity exists between these factors and other multiple factors measured in our study (e.g., body mass index, blood pressure and cholesterol level), which were considered in the multivariable analyses, is seems unlikely that these two factors would introduce a systematic bias on the vaccination effectiveness estimates.

Given the large size of our study population, many statistically significant differences appear when comparing vaccinated and unvaccinated groups, but most of them were not substantial. The authors performed multivariable adjustment for potential confounders to account for these differences in the statistical analysis. However, as with all observational studies, the possible influence of residual confounding or a healthy user effect on the estimates of vaccine effectiveness cannot be completely excluded considering that vaccination was non-randomized.

The relatively high proportion of unvaccinated subjects in the present cohort study (67%) is largely due to criteria used to define cohort members as adequately immunized against pneumococcus (at least one dose of PPV23 within 5 years prior study start). According this restrictive definition, many elderly individuals (without criteria for revaccination) were classified as unvaccinated because they had received PPV23 more than five years before study start and this fact contributed to the relatively high size of the unvaccinated group. If we consider all individuals who had received a dose of PPV23 at any time, the proportion of cohort members never vaccinated against pneumococcus would be only 44%, which fits with data reported in other studies assessing PPV effectiveness [6].

On other hand, it must be noted that criteria used to classify individuals as adequately immunized against pneumococcus (at least one dose of PPV23 in prior 60 months before study start) can impact the results evaluating vaccination effectiveness. Although it has been reported that antibody level declines 3-5 years after vaccination, it is possible that a protective effect of PPV23 (if exists) could remain over time.

Future analyses (involving a considerable higher number of person-years followed) should evaluate the effects of the vaccine according to time elapsed since vaccination. These analyses should investigate the possible vaccination effectiveness among those individuals who received the PPV23 in prior 6-10 years as compared with those who received the vaccine in prior 5 years or those who were never vaccinated.

Given the demonstrated efficacy of PPV23 against invasive pneumococcal disease, commencing new Randomized Controlled Trials in populations at risk would create ethical difficulties. Thus, prospective cohort studies using validated clinical data are an acceptable alternative to estimate vaccine effectiveness against different clinically relevant outcomes among different populations at risk.

## Conclusions

We emphasise that this study should be considered as an interim analysis. A further detailed analysis, including more person-years of observation, should provide accurate population-based data on incidence of myocardial infarction and stroke in different subsets of the population in order to establish, if confirms, the magnitude of vaccination effectiveness in primary and/or secondary cardiovascular prevention.

### Support statement and role of the funding source

This work was supported by a grant from the "Fondo de Investigación Sanitaria" of the Instituto de Salud Carlos III [FIS 09/00043] of the Spanish Healh Ministry.

This study sponsor had no role in the design or implementation of the study, analysis of data, or reporting of the results.

## Competing interests

The authors declare that they have no competing interests.

## Authors' contributions

AV-C and OO-G designed the study, assessed outcomes, and wrote and edited the paper; AG-P, AV-R, FG, XR, ES, CdD and ES obtained the data; TR-B did statistical analysis, AV-C coordinated the study. All authors read and approved the final manuscript.

## Pre-publication history

The pre-publication history for this paper can be accessed here:

http://www.biomedcentral.com/1471-2458/12/222/prepub
